# Lipoprotein(a), Endothelial Dysfunction and Cardiovascular Disease

**DOI:** 10.1111/jcmm.71426

**Published:** 2026-11-06

**Authors:** Xianghui Zhou, Xiyuan Fang, Xin Zhou, Zhao Zhang, Yu Hu, Zhipeng Cheng

**Affiliations:** ^1^ Department of Hematology Union Hospital, Tongji Medical College, Huazhong University of Science and Technology Wuhan China; ^2^ Department of Stomatology Union Hospital, Tongji Medical College, Huazhong University of Science and Technology Wuhan China; ^3^ Hubei Clinical Medical Center of Cell Therapy for Neoplastic Disease Wuhan China

**Keywords:** cardiovascular disease, endothelial cells, lipoprotein(a)

## Abstract

Lipoprotein(a) (Lp(a)) is a particle similar to low‐density lipoprotein cholesterol and is associated with apolipoprotein (A). High Lp(a) levels are a genetic, independent risk factor for atherosclerotic cardiovascular disease (ASCVD). Endothelial cells, which form the continuous lining of the cardiovascular system, are crucial for vascular function and represent the primary target of Lp(a)‐mediated injury. Lp(a) penetrates the endothelial barrier and undergoes oxidative modification, triggering endothelial dysfunction, which is characterized by inflammation, oxidative stress and impaired nitric oxide bioavailability. These disruptions promote monocyte recruitment, foam cell formation and atherosclerosis development. Currently, various drugs that can reduce Lp(a) levels are also involved in regulating endothelial cell function, ultimately reducing the risk of ASCVD. However, the molecular mechanisms underlying Lp(a)‐mediated endothelial injury and the extent to which Lp(a) lowering improves endothelial function remain incompletely defined. Clarifying these issues may inform cardiovascular risk stratification and the clinical development of Lp(a)‐targeted therapies. This review seeks to consolidate the understanding of the link between Lp(a) levels and endothelial dysfunction, highlighting its pivotal role in cardiovascular disease progression.

## 1 Introduction

The incidence of and mortality from cardiovascular diseases remain high worldwide. The 2019 World Health Organization data indicate that cardiovascular and cerebrovascular diseases caused approximately 17.9 million deaths, accounting for 32% of the total mortality rate. This trend is estimated to continue, with the annual death count increasing to nearly 24 million by 2030 (https://www.who.int/) [1]. Atherosclerosis contributes to the onset and progression of cardiovascular diseases. Studies have shown that during atherosclerosis progression, lipids accumulate in blood vessel walls, triggering inflammatory responses; these responses cause lipid metabolism imbalance and immune responses, leading to chronic inflammation of the arterial walls [2]. Lipids play crucial roles in the development of atherosclerosis. Since 1963, researchers have identified lipoprotein(a) (Lp(a)) in plasma, a unique lipoprotein resembling low‐density lipoprotein (LDL). Lp(a) can penetrate the endothelium, accumulate in the subendothelial space, and potentially promote atherosclerosis development [3, 4]. Clinical studies have indicated that elevated Lp(a) levels are an independent, heritable risk factor for cardiovascular disease (CVD). Furthermore, evidence suggests that high plasma Lp(a) levels contribute to faster CVD progression and higher cardiovascular mortality [5]. The exact mechanism through which Lp(a) influences cardiovascular disease progression is not yet understood.

In atherosclerosis, endothelial cell dysfunction is the earliest detectable change [6]. Normal endothelial cells constitute the barrier separating blood from the surrounding tissues. Through their structure and various functions, endothelial cells mediate important physiological processes, such as maintaining vascular permeability, coagulation, metabolism, and immunity [7]. In the initial phase of atherosclerosis, endothelial cells transition from a quiescent state to an activated state because of the influence of proinflammatory cytokines and turbulent blood flow [8]. Neutrophils and monocytes are recruited into the intima, and the monocytes then differentiate into macrophages. Endothelial cells and macrophages produce diverse chemokines and growth factors. Various cells interact with each other, exacerbating local inflammation and causing the continuous progression of plaques [9].

Both plasma Lp(a) levels and endothelial cell dysfunction are significant risk factors for the progression of cardiovascular disease and atherosclerosis. Research has indicated that Lp(a) can modify the inflammatory environment, change monocyte phenotypes, and enhance thrombosis [10, 11, 12]. Recent reviews have comprehensively summarised the broader proatherogenic, proinflammatory, and prothrombotic effects of Lp(a) across multiple vascular cell types [13]. In contrast, the present review focuses specifically on the vascular endothelium as the interface linking circulating Lp(a) to arterial‐wall injury. We delineate the synthesis and metabolism of Lp(a) and integrate evidence on endothelial transcytosis and subendothelial retention, inflammatory signalling, eNOS dysfunction, oxidative stress, pyroptosis, and metabolic reprogramming. Additionally, we summarise and discuss the scientific literature on the relationship between Lp(a) and endothelial cells and its relationship with cardiovascular diseases and provide future directions to explore in this field.

## 2 Lipoprotein(a): Overview of Structure, Synthesis, and Metabolism

In terms of its structure, lipoprotein(a) is similar to LDL cholesterol (LDL‐C) but different from LDL‐C. It consists of a lipid core made of cholesteryl esters and triglycerides, which is surrounded by an outer shell of phospholipids, free cholesterol, and apolipoprotein B‐100 (apoB‐100) particles [14, 15]. Unlike LDL‐C, it includes an extra glycoprotein, namely apolipoprotein(a). Apolipoprotein(a) is structurally linked to apo‐B100 through a disulfide thioester bond, altering its molecular weight and density compared with LDL‐C [14]. It is encoded by the *LPA* gene located at positions 26 and 27 on the long arm of chromosome 6 (6q26–27) [16]. Apolipoprotein(a) is highly polymorphic and contains a unique protein domain called ‘kringle’, which contains a variable number of cysteine‐rich domains. The gene encoding apolipoprotein(a) exhibits up to 70% homology with the plasminogen gene, and its structure is similar to that of plasminogen [17]. Plasminogen consists of five kringle protein domains (KI–KV) and a protease domain. In contrast, apolipoprotein(a) comprises a kringle V domain (KV), 10 distinct kringle IV domains (KIV1–KIV10), and an inactive protease domain [18]. The KIV domain typically exists as a single copy, whereas the KIV2 domain varies in copy number, ranging from 2 to > 40, influencing the size of apolipoprotein(a). Single‐nucleotide polymorphisms (SNPs) in the *LPA* gene influence the height variation of apolipoprotein(a) [19, 20, 21, 22].

Lp(a) is synthesized and secreted mainly by liver cells, as indicated by the presence of significant levels of apolipoprotein(a) mRNA in human hepatocytes. The synthesis of Lp(a) involves three stages: transcription of the apo(a) gene, translation, and necessary posttranslational modifications for apolipoprotein(a) assembly [23]. It initially undergoes posttranslational modifications in the endoplasmic reticulum, including the formation of disulfide bonds within each kringle motif and the addition of N‐ and O‐linked glycans [24]. Interestingly, the time required to exit the endoplasmic reticulum varies with the kringle number. A longer residence time in the endoplasmic reticulum has been found for larger apo(a) isoforms; thus, they are more prone to degradation through the endoplasmic reticulum‐associated pathway. This also explains why circulating Lp(a) levels are roughly proportional to the size of the apo(a) isoform. These findings suggest that the primary factor influencing plasma Lp(a) levels is Lp(a) synthesis [25]. The formation of Lp(a) likely involves a two‐step process combining LDL and apo(a). Initially, apo(a) binds to LDL via specific interactions between weak lysine binding sites in the KIV7 and KIV8 domains of apolipoprotein(a) and particular lysine residues in the amino‐terminal domain of apoB‐100. In the second step, a disulfide bridge forms, stabilizing the mature Lp(a) [26]. The location of Lp(a) assembly remains unclear. Hepatocyte‐based studies support extracellular assembly at the cell surface or within the space of Disse, whereas human kinetic studies are compatible with intracellular assembly before secretion. These findings may reflect complementary pathways rather than mutually exclusive mechanisms, although direct evidence currently favours extracellular assembly near the hepatocyte surface. Defining their relative contributions may inform therapies targeting apo(a)–apoB association [27, 28].

Lp(a) molecules are mainly cleared by the liver, with minor contributions from the spleen and kidneys. The detailed mechanism of Lp(a) catabolism by the liver remains unclear, but the asialoglycoprotein receptor is thought to contribute to the clearance of a small fraction of Lp(a) [26]. Various cell surface receptors, such as scavenger receptors, carbohydrate receptors, plasminogen receptors, Toll‐like receptors (TLRs), and several LDL receptors (LDL‐Rs), have been suggested to play roles in Lp(a) clearance [29]. Research has extensively explored the role of LDL‐R in Lp(a) uptake; the results have indicated that PCSK9 modulates the internalization of Lp(a) by hepatocytes through LDL‐R [30]. The significant homology between apolipoprotein(a) and plasminogen has led to increased research interest in the role of plasminogen receptors in Lp(a) metabolism. Studies have indicated that the plasminogen receptor KT (Plg‐RKT) is crucial for Lp(a) uptake. Following Lp(a) internalization, the apo(a) component is directed to recycling endosomes, which aid in the re‐secretion of the protein. Sharma M's research group discovered that reducing Plg‐RKT expression in haploid fibroblast‐like HAP1 cells decreased Lp(a) uptake. Similarly, the use of Plg‐RKT‐specific siRNA to knock down the receptor in HepG2 cells led to marked decreases in Lp(a) uptake. Plg‐RKT overexpression led to marked increases in Lp(a) uptake in both cell lines [31]. Despite numerous studies on Lp(a) synthesis and metabolism, conflicting results and unclear mechanisms indicate the need for further research (Figure 1).

**FIGURE 1:**
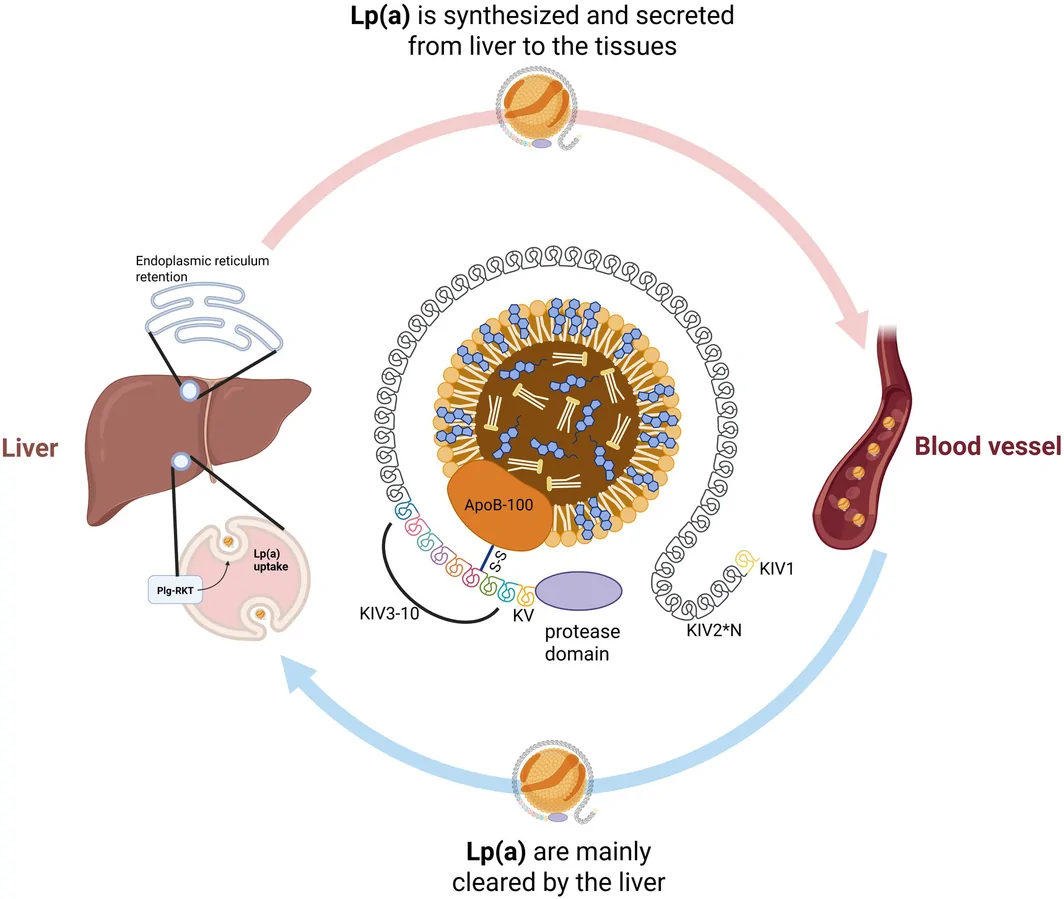
The structure, synthesis and metabolism of Lp(a). Lp(a) consists of a lipid core made of cholesterol esters and triglycerides, surrounded by an outer shell of phospholipids, free cholesterol and apolipoprotein B‐100 (apoB‐100) particles. It is synthesized mainly in hepatocytes, where apo(a) undergoes endoplasmic reticulum retention. Secreted Lp(a) enters the circulation and is cleared primarily by the liver through multiple candidate receptors, including lipoprotein, scavenger, and plasminogen receptors such as Plg‐RKT.

## 3 Endothelial Cell Function and Cardiovascular Disease

Endothelial cells form the inner lining of all blood vessels and play a crucial role in maintaining cardiovascular homeostasis. In the healthy state, the vascular endothelium serves as a mechanical barrier and an autocrine and paracrine organ, supporting the normal functioning of tissues and organs. Research has indicated that the endothelium supports vascular elasticity, supplies oxygen and nutrients to tissues, prevents platelet aggregation, and has roles in anticoagulation, profibrinolysis, and the inhibition of smooth muscle cell proliferation and migration. Endothelial cell functional homeostasis plays important protective roles in atherosclerosis, limiting inflammation, inhibiting leukocyte adhesion and transport, and reducing vascular permeability [32]. The maintenance of these basic physiological processes depends on the mutual regulation between endothelial cells and other functional tissue cells, such as smooth muscle cells, monocytes, and macrophages. Under an imbalance of the regulatory homeostasis between endothelial cells and other tissue cells, endothelial cell dysfunction occurs. This imbalance tends to lead to proinflammatory, prothrombotic, and proatherosclerotic phenotypes in the vascular system, which result in vascular dysfunction and pathological remodelling, ultimately leading to cardiovascular disease development [33]. (Figure 2) Considering the comprehensive insights into the varied roles of endothelial dysfunction in cardiovascular disease, we succinctly examine its significant role in atherosclerosis.

**FIGURE 2:**
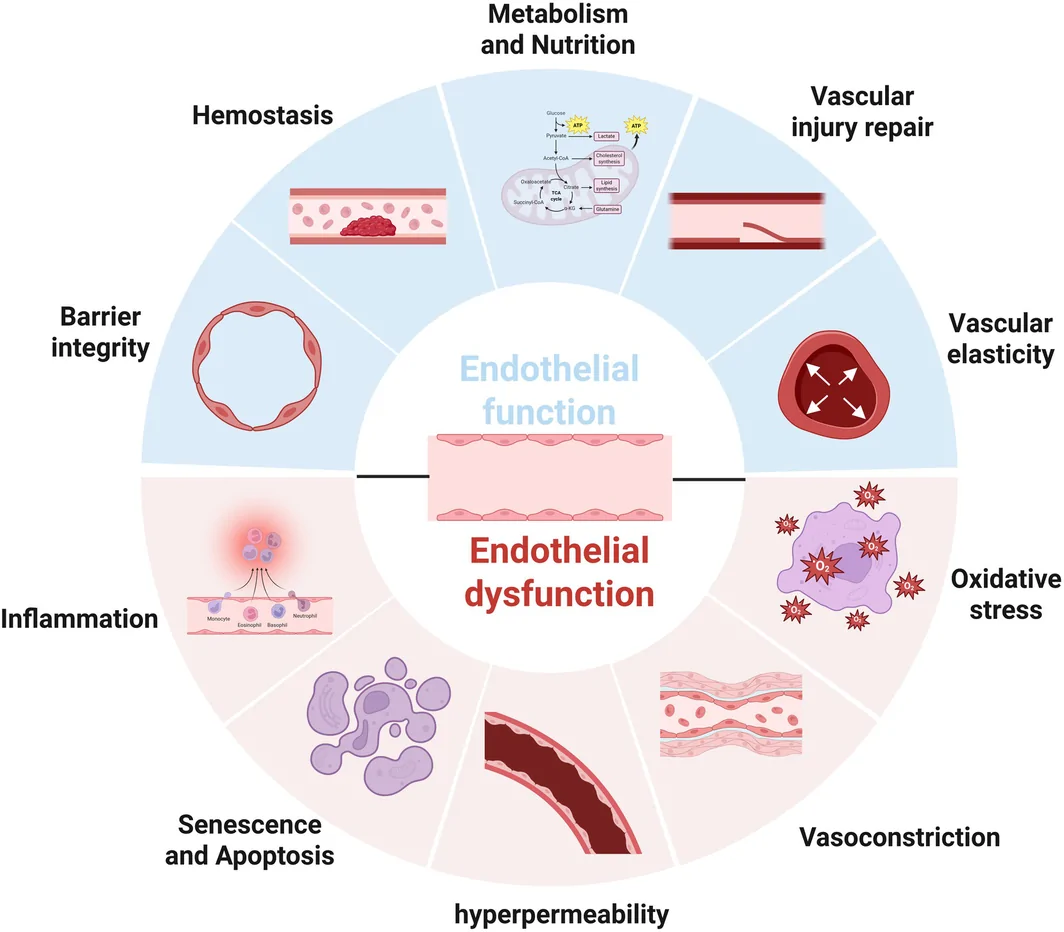
Endothelial function and endothelial dysfunction. Normal endothelial cells possess a variety of biological functions, including maintaining the integrity of blood vessels; acting as a semi‐permeable membrane barrier to regulate metabolism and nutrient transport; repairing vascular injuries; regulating thrombosis and haemostasis; and regulating vascular elasticity, etc. The characteristic manifestations of endothelial dysfunction include an inflammatory state, high permeability, oxidative stress, vasoconstriction, cellular senescence and apoptosis.

### 3.1 Endothelial Barrier and Atherosclerosis

The vascular endothelium, the innermost layer of blood vessels, serves as a semipermeable barrier that regulates the movement of fluids, ions, molecules, and leukocytes between the lumen and the external environment [34]. Intercellular junctions preserve endothelial integrity, preventing vascular leakage [35]. These cell–cell junctions contain protein complexes from adherens, gap, and tight junctions, along with other adhesion receptors such as CD31/PECAM‐1. However, long‐term inflammation, oxidative stress, ischaemia and trauma and other inducing factors lead to abnormal barrier function; hence, leukocytes in the vessels can infiltrate the blood vessel wall, thereby aggravating the inflammatory state and promoting atherosclerosis [36].

### 3.2 Endothelial Proinflammatory State and Atherosclerosis

Recent research into immune regulation in atherosclerosis has revealed that it is a chronic inflammatory disease in which endothelial cells play a crucial role. In healthy resting vasculature, endothelial cells maintain vascular homeostasis by minimizing inflammation, thrombosis, and permeability. Upon stimulation, endothelial cells alter their functional phenotype by expressing effector proteins crucial for immune cell recruitment and new vessel formation, which has significant pathophysiologic implications [37]. Endothelial proinflammatory phenotypes induced by stimulation can be divided into two categories: some inflammatory ligands induce rapid and transient endothelial cell responses, known as Type I endothelial activation, whereas other inflammatory ligands mediate persistent endothelial cell responses involving the induction of new genes, known as Type II endothelial activation [38, 39].

### 3.3 Flow‐Mediated Endothelial Function in Atherosclerosis

Haemodynamic force variations due to flow affect the endothelial phenotype induced and regulate vascular processes, such as acute vessel tone maintenance, vascular permeability, leukocyte adhesion, blood vessel development, and the secretion of prothrombotic and antithrombotic signalling molecules. In the straight sections of arteries, laminar blood flow and high wall shear stress contribute to an atheroprotective effect, supporting endothelial cell function [40]. In contrast, in the branches and curvatures of arteries, where blood flow is disrupted and wall shear stress is uneven and irregularly low, atherogenesis may be facilitated, contributing to conditions such as atherosclerosis and peripheral artery disease [41, 42]. Laminar shear stress restricts proinflammatory gene expression and sustains endothelial cell quiescence through eNOS activation or KLF2 expression. Oscillatory shear stress facilitates leukocyte recruitment to inflammatory sites, where they adhere to vessel walls through interactions with adhesion molecules, contributing to atherosclerosis development. Blood flow turbulence can induce endothelial cell remodelling and increase fibronectin expression, resulting in cytoskeletal rearrangement and endothelial barrier dysfunction [43, 44, 45, 46].

## 4 Influence of Lp(a) on Endothelial Function

### 4.1 Endothelial Penetration and Subendothelial Retention

The initial and obligatory step in Lp(a)‐mediated atherogenesis is its transit across the endothelial barrier into the subendothelial space, followed by its retention within the extracellular matrix (ECM). This process transforms Lp(a) from a circulating lipoprotein into a sustained local inflammatory stimulus.

Lp(a) crosses the endothelial barrier through active receptor‐mediated transcytosis rather than passive leakage. Henry et al. demonstrated that Lp(a) undergoes activin receptor‐like kinase 1 (ALK1)‐ and caveolin‐1 (CAV1)‐dependent transcytosis in human coronary artery endothelial cells (HCAECs). ALK1 or CAV1 knockdown significantly attenuated this process, whereas LDL‐R or SR‐BI knockdown had no effect. Anti‐ALK1 antibodies reduced Lp(a) transcytosis and Lp(a)‐induced, but not LDL‐induced, SMAD1/5 phosphorylation downstream of ALK1. However, direct binding of Lp(a) to ALK1 and the requirement for canonical ALK1–SMAD1/5 signalling have not been established. Because ALK1 also mediates LDL transcytosis independently of its kinase activity, ALK1‐dependent transport is not specific to Lp(a). Further studies are required to define the contribution of the apo(a) moiety and the signalling dependence of this process [47, 48, 49].

Additionally, the apolipoprotein(a) moiety of Lp(a) can lead to an increase in myosin light chain (MLC) phosphorylation, triggering the reorganisation of the actin cytoskeleton, inducing the formation of F‐actin stress fibres, and disrupting the contact between endothelial cells, all of which increase endothelial permeability and facilitate the internalisation of lipoproteins [50]. Mechanistically, the endothelial barrier dysfunction induced by apo(a) is mediated through the Rho/Rho kinase‐dependent pathway, which results in increased MYPT1 phosphorylation. This reduces MLC phosphatase activity, thereby increasing MLC phosphorylation, stress fibre formation, cell shrinkage, and endothelial permeability [51].

Once in the subendothelial space, Lp(a) is retained through high‐affinity interactions with the subendothelial ECM (a dense mesh formed of collagens, fibronectin, laminin, and proteoglycans). Pillarisetti et al. used heparinase to remove heparan sulfate proteoglycans (HSPGs), which increased Lp(a) binding to the subendothelial matrix by 2–10‐fold. Anti‐fibronectin antibodies inhibited this increase by 60%, indicating that fibronectin is the critical binding partner that gets exposed after HSPG removal. Ox‐LDL‐treated endothelial cells produced heparanase‐like activity, which reduced HSPG levels and promoted Lp(a) retention [52]. At the molecular level, recombinant apo(a) bound strongly to fibronectin and fibrinogen, weakly to laminin, and not at all to von Willebrand factor or vitronectin [53]. PLA2 modification enhanced Lp(a) retention in the subendothelial matrix by fourfold, with enhanced binding to fibronectin, laminin, and collagen [54]. Furthermore, an analysis revealed that proteoglycans rich in chondroitin sulfate have a high affinity for Lp(a), reflecting the role of Lp(a) in arterial lesions [55].

Retained Lp(a) is highly susceptible to oxidative modification. Lp(a) is the primary plasma carrier of oxidised phospholipids (ox‐PL). ox‐PL colocalise with apo(a)‐Lp(a) in arterial lesions and directly promote endothelial dysfunction, lipid deposition, and inflammation [56]. Furthermore, studies have shown that ox‐Lp(a) promotes the adhesion of monocytes to endothelial cells. Northern blot analysis revealed that ox‐Lp(a) induces the expression of P‐selectin in human umbilical vein endothelial cells (HUVECs) [57]. This oxidative modification creates a self‐perpetuating cycle. Retained Lp(a) undergoes oxidation, mediating the release of proinflammatory mediators that recruit inflammatory cells; this process in turn leads to the production of more oxidants, amplifying local inflammation and resulting in downstream endothelial activation and atherosclerotic plaque formation [58].

### 4.2 Lp(a)‐Induced Inflammatory Activation of Endothelial Cells

The inflammatory activation of endothelial cells represents a central mechanism through which Lp(a) initiates atherosclerosis and enhances its progression. Lp(a) binds to endothelial cells through multiple receptor‐mediated interactions, triggering a coordinated inflammatory response involving intracellular signalling cascades, transcriptional upregulation of proinflammatory mediators, amplification of endothelial activation, and promotion of further leukocyte recruitment. The inflammatory cascade triggered by Lp(a) translates into quantifiable vascular inflammation in humans. Van der Valk et al. used ^[18F]^FDG PET/CT imaging to demonstrate that subjects with elevated Lp(a) exhibited significantly increased arterial wall inflammation in the carotid arteries and ascending aorta, quantified as an elevated maximum target‐to‐background ratio (TBR), compared with controls. These findings provide direct in vivo evidence linking Lp(a)‐induced endothelial activation to detectable arterial inflammation [59].

Previous studies have reported that the inflammatory cascade triggered by Lp(a) in endothelial cells is regulated mainly through two signalling pathways: the nuclear factor κB (NF‐κB) pathway and the mitogen‐activated protein kinase (MAPK) pathway [60]. In HCAECs, Lp(a) stimulation significantly increases the expression of NF‐κB p65, the transcriptionally active subunit of the NF‐κB complex. This upregulation reflects the transcriptional activation of the NF‐κB pathway, indicating that this pathway is a critical mediator of the proinflammatory effects of Lp(a) on the endothelium [61]. Mechanistically, Lp(a) activates these signalling cascades through interactions with multiple cell surface receptors. Lp(a) binds to Toll‐like receptor 2 (TLR2) and TLR4, along with the coreceptors CD14 and CD36, which collectively funnel into NF‐κB‐dependent inflammatory signalling [62]. In addition, evidence indicates that the apo(a) component of Lp(a) activates endothelial cells through integrin αVβ3 signalling, leading to the increased phosphorylation of focal adhesion kinase (FAK) and MAPKs such as extracellular signal‐regulated kinase (ERK), p38, and c‐Jun N‐terminal kinase (JNK) [63]. In synergy with NF‐κB, the MAPK pathway amplifies and sustains the inflammatory response. Lp(a) activates monocytes and endothelial cells through the MAPK signalling cascade, promoting inflammation in a manner that is integrated with NF‐κB‐dependent transcriptional programmes. The convergence of these two pathways (NF‐κB and MAPK) ensures a robust and sustained proinflammatory signal that drives the downstream activation of endothelial cells.

Following the activation of NF‐κB and MAPK signalling, Lp(a) enhances the expression of two major classes of proinflammatory mediators: adhesion molecules and inflammatory cytokines. Takami's research group showed that Lp(a) increases both the mRNA and cell surface expression levels of ICAM‐1 in endothelial cells, thereby enhancing the inflammatory response and contributing to cardiovascular disease progression [64]. Research has indicated that Lp(a) increases the expression of vascular cell adhesion molecule 1 (VCAM‐1) and E‐selectin in HCAECs by increasing intracellular free calcium levels [65]. The biochemical analysis of full‐thickness aortic wall tissues from patients with atherosclerosis revealed that the plasma Lp(a) level was positively correlated with the expression of VCAM‐1 in aortic wall tissues; this result also suggests that Lp(a) is involved in the secretion of adhesion molecules [66]. Concurrently, Lp(a) induces the expression of inflammatory cytokines, including interleukin (IL)‐1β, IL‐6, IL‐8, and tumour necrosis factor‐α (TNF‐α) [62]. Evidence has indicated that Lp(a) leads to the increased expression of monocyte chemoattractant protein‐1 (MCP‐1) [67]. Specifically, in HCAECs, Lp(a) stimulation significantly increases IL‐6 expression at the protein level [61]. Other studies have shown that Lp(a) induces HUVECs to secrete monocyte chemotactic activity (MCA), promotes the recruitment of monocytes to the vascular wall, and contributes to the occurrence of atherosclerosis [68]. Research has also suggested that the monocyte chemotactic activity stimulated by Lp(a) in human vascular endothelial cells may be closely related to the chemokine I‐309. Through immunohistochemistry, I‐309 was detected in endothelial cells, macrophages, and extracellular regions of human atherosclerotic plaques, and was found to colocalise with apo(a), indicating that it is involved in the Lp(a)‐stimulated monocyte chemotactic activity of human vascular endothelial cells [69].

These adhesion molecules play unique roles in the leukocyte adhesion cascade, and the continuous inflammatory response, in turn, aggravates endothelial function and enhances lipid retention, thereby promoting plaque growth.

### 4.3 Lp(a)‐Induced eNOS Dysfunction and Oxidative Stress

Endothelial nitric oxide synthase (eNOS) dysfunction and oxidative stress are closely linked pathological processes that constitute a core mechanism contributing to Lp(a)‐induced vascular injury. The functional integrity of eNOS is essential for maintaining vascular homeostasis through the production of nitric oxide (NO), a potent vasodilator with anti‐inflammatory, antithrombotic, and antiproliferative properties [70]. Lp(a) disrupts this protective system through multiple converging mechanisms, establishing a pro‐oxidative, proinflammatory state that predisposes patients to atherosclerosis.

Experimental evidence has indicated that Lp(a) stimulation significantly reduces eNOS expression at the protein level in HCAECs [61]. This downregulated expression represents a fundamental disruption of endothelial function, as reduced eNOS levels directly translate to decreased NO production. The association between elevated Lp(a) and impaired endothelium‐dependent vasodilation has been demonstrated in human studies. Tsurumi's clinical research on the diameter of coronary arteries revealed a significant negative correlation between Lp(a) and the arterial vascular response [71]. Wu et al. examined 89 healthy subjects and found that plasma Lp(a) levels correlated inversely with flow‐mediated dilation (FMD) of the brachial artery, a well‐established measure of endothelial function. This observation indicates that Lp(a) selectively impairs NO‐dependent vasomotor function [72]. Bolen et al. prospectively evaluated coronary flow reserve (CFR) in 25 patients with elevated Lp(a) and 30 controls. CFR was significantly lower in the elevated Lp(a) group, with Lp(a) levels inversely correlating with CFR. These findings provide direct clinical evidence that elevated Lp(a) compromises coronary vasomotor function [73].

The most significant mechanism through which Lp(a) impairs eNOS function is eNOS uncoupling, a pathological state in which the enzyme generates superoxide (O_2_^−^). Polkinghorne et al. provided the first human evidence indicating that the genetically determined elevation of plasma Lp(a) results in dysregulated vascular redox/nitrosative signalling. In 1027 patients with advanced coronary artery disease, increased plasma Lp(a) levels were associated with elevated arterial superoxide levels. Crucially, this effect was primarily due to eNOS uncoupling through the reduced bioavailability of vascular BH4 (tetrahydrobiopterin). BH4 bioavailability is regulated by GTPCH1‐dependent de novo synthesis, oxidative loss, and DHFR‐mediated recycling of BH2 to BH4. Oxidative stress, particularly peroxynitrite formation, promotes BH4 oxidation and lowers the BH4:BH2 ratio, whereas reduced GTPCH1 activity or impaired DHFR‐dependent recycling further limits BH4 availability. The resulting eNOS uncoupling decreases NO production and increases superoxide generation, thereby amplifying oxidative stress and further promoting BH4 oxidation. However, whether Lp(a) directly regulates GTPCH1 or DHFR remains to be established. Variability in Lp(a) levels did not significantly affect vascular NOX‐derived superoxide levels, indicating that eNOS uncoupling is the principal mechanism of Lp(a)‐mediated oxidative stress [74, 75, 76].

Beyond the eNOS uncoupling mechanism, Lp(a) triggers oxidative stress through additional mechanisms. Lp(a) stimulates endothelial cells to produce reactive oxygen species (ROS), contributing to endothelial dysfunction [62]. Lp(a) carrying ox‐PL is responsible for proinflammatory responses in endothelial cells [67]. ROS generation is a direct consequence of Lp(a) interactions with the endothelium. Evidence indicates that ox‐Lp(a) may enhance the permeability of the endothelial cell monolayer by increasing ROS generation. The mechanism may involve ox‐Lp(a) suppressing desmoglein‐1 (DSG1) and desmocollin‐2 (DSC2) expression at both the mRNA and protein levels, thereby promoting cellular ROS generation [77]. Additionally, Iwabayashi et al. reported that Lp(a) accelerates the ageing of human aortic endothelial cells by upregulating p53 and p21 expression, which induces excessive ROS generation, contributing to atherosclerosis development [78]. Furthermore, ox‐Lp(a) triggers autophagy in HUVECs through ROS generation; this effect of ox‐Lp(a) involves the LKB1‐AMPK‐mTOR and PAPR‐1–LKB1–AMPK–mTOR pathways [79].

Thus, Lp(a) induces eNOS dysfunction and oxidative stress through a triad of mechanisms—direct downregulation of eNOS expression, eNOS uncoupling through reduced BH4 bioavailability, and induction of ROS that further amplifies injury, all of which converge to establish a proatherogenic endothelial phenotype.

### 4.4 Emerging Mechanisms: Pyroptosis and Metabolic Reprogramming

Recent research has identified two additional mechanisms through which Lp(a) induces endothelial injury: pyroptosis, a proinflammatory form of programmed cell death, and metabolic reprogramming, which is characterised by a shift toward glycolysis.

Pyroptosis is a distinct form of lytic cell death that is inherently proinflammatory and is characterised by cell swelling, membrane rupture, and the release of intracellular contents that amplify local inflammation [80]. Cao et al. demonstrated that ox‐Lp(a) significantly increases the expression of pyroptosis‐related proteins (NLRP3, pro‐caspase‐1, caspase‐1, GSDMD and GSDMD‐N) in HUVECs, confirming the occurrence of pyroptotic cell death. The mechanism involves mitochondrial dysfunction through cytochrome b (CYTB) downregulation, which is accompanied by increased mitochondrial ROS levels, Ca^2+^ overload, decreased ATP levels, and abnormal mitochondrial morphology. CYTB overexpression partially reverses these effects; this observation indicates that ox‐Lp(a) induces pyroptosis through CYTB downregulation. Given that Lp(a) is the primary carrier of ox‐PL and is highly susceptible to oxidation, ox‐Lp(a)‐induced pyroptosis represents a novel pathway linking Lp(a) oxidation to endothelial cell dysfunction and vascular inflammation [81].

Complementing pyroptosis, Lp(a) can induce a proinflammatory state in endothelial cells through the upregulation of key glycolytic players, such as glucose transporter 1 and HK. Glycolysis is a series of reactions in which glucose is degraded into pyruvate and ATP is generated. It can occur under both anaerobic and aerobic conditions, and endothelial cells rely mainly on glycolysis as their main energy source. Many studies have revealed changes in endothelial cell metabolism in atherosclerotic lesions, along with significant increases in the rate of glycolysis. Johanhan G revealed that 6‐phosphofructo‐2‐kinase/fructose‐2,6‐bisphosphatase (PFKFB3) may play an important role in Lp(a)‐induced endothelial cell inflammation, and this process is also driven by glycolysis. The researchers showed that in response to the glycolysis activator PFKFB3, the glycolytic activity of endothelial cells containing Lp(a) is significantly increased, and the inhibition of PFKFB3 not only alleviates the increase in endothelial cell glycolysis induced by Lp(a) but also inhibits the inflammatory response and reduces the secretion of IL‐6 and MCP‐1 by endothelial cells. The inhibition or loss of PFKFB3 further affects the migration function of endothelial cells, which depends on the remodelling of the actin cytoskeleton. In addition, the study emphasised the importance of ox‐Lp(a) in mediating proinflammatory responses. Lp(a) mainly promotes glycolysis in endothelial cells through its ox‐PL activity, thereby driving the proinflammatory process and affecting endothelial cell homeostasis [82, 83].

Thus, these emerging pathways expand our understanding of Lp(a)‐mediated endothelial injury beyond classical inflammation and oxidative stress, revealing new mechanistic links between Lp(a) oxidation, metabolic dysregulation, and endothelial cell loss, all of which collectively promote the development of atherosclerotic lesions.

### 4.5 Impaired Endothelial Fibrinolysis and Potential Thrombotic Implications

The apo(a) component of Lp(a) shares structural homology with plasminogen but lacks its proteolytic activity. Experimental studies show that Lp(a) can inhibit plasminogen binding to endothelial cells and reduce plasmin generation, potentially impairing local fibrinolysis [84]. Endothelial surface receptors organise plasminogen activation; receptor‐manipulation studies implicate urokinase‐type plasminogen activator receptor (uPAR) and, to a lesser extent, selected integrins in apo(a)‐mediated inhibition of this process [85]. These findings link Lp(a) to disruption of the endothelial fibrinolytic surface.

Nevertheless, the clinical contribution of these antifibrinolytic effects remains uncertain. A small randomised study found that substantial antisense‐mediated Lp(a) lowering did not significantly alter ex vivo plasma clot lysis [86]. Thus, impaired endothelial fibrinolysis represents a plausible contributor to atherothrombosis, but its importance in humans and reversibility with Lp(a)‐targeted treatment require further investigation.

## 5 Association of Lp(a) Reduction Strategies with Endothelial Cells

Recent research has shown that the plasma levels of Lp(a) can be reduced through various methods, such as lipid‐lowering drugs initially designed for lowering LDL cholesterol, therapies specifically targeting Lp(a) (small‐molecule inhibitors, antisense oligonucleotides, etc.), and gene editing therapies. The link between Lp(a) levels and endothelial function has important implications for CVD prevention. In the following section, we introduce these therapies related to endothelial cell function.

Oestrogen‐related drugs have been reported to significantly reduce Lp(a) levels. A recent meta‐analysis revealed that medroxyprogesterone acetate combined with conjugated equine oestrogens (MPA/CEE) reduces lipoprotein(a) levels in postmenopausal women, suggesting a reduced cardiovascular disease risk [87]. A study was conducted on 27,736 healthy women to investigate whether hormone replacement therapy (HT) affects Lp(a) levels. The women who underwent HT were divided into those who were taking only oestrogen and those who were taking a combination of oestrogen and progesterone. Lp(a) levels were lower in women undergoing HT [88]. A stable‐isotope kinetic study demonstrated that oestrogen treatment reduced plasma Lp(a) concentrations by approximately 20% and the apo(a) production rate by 34%, without significantly altering the fractional catabolic rate of apo(a), suggesting that oestrogen lowers Lp(a) primarily by reducing its production rather than accelerating its clearance [89]. Moreover, studies have demonstrated that oestrogen deficiency caused by oophorectomy led to increases in serum Lp(a) levels, whereas 17β‐estradiol replacement therapy reversed this change. In cultured endothelial cells, pretreatment with oestrogen attenuated Lp(a)‐induced increases in the mRNA expression levels of ICAM‐1, VCAM‐1, E‐selectin and MCP‐1. These findings suggest that oestrogen can protect against CVD development by regulating endothelial function [90].

Nicotinic acid is the precursor of nicotinamide adenine dinucleotide; thus, it participates in the regulation of various cellular functions, including redox reactions and the maintenance of stress resistance. Currently, niacin is commonly used to treat lipid disorders and cardiovascular diseases [91]. Numerous clinical trials have demonstrated that niacin therapy, whether used independently or alongside other lipid‐lowering medications, results in significant decreases in overall mortality and coronary artery events while also delaying and potentially reversing coronary atherosclerosis [92, 93]. Charles et al. confirmed that nicotinic acid can reduce plasma Lp(a) levels [94]. Their study investigated the relationships between dietary niacin intake and both vascular endothelial function and oxidative stress in healthy middle‐aged and elderly individuals. A study in 127 individuals aged 48–77 years revealed a negative correlation between plasma oxidized low‐density lipoprotein levels and niacin intake. A meta‐analysis suggests that nicotinic acid typically reduces circulating Lp(a) levels by approximately 20%–30% [95]. This effect is probably mediated, at least in part, by suppression of hepatic LPA transcription and reduction of apo(a) production or secretion [96]. Furthermore, increased dietary niacin intake is linked to improved vascular endothelial function and reduced systemic and vascular oxidative stress [97].

Eicosapentaenoic acid (EPA), a highly unsaturated omega‐3 fatty acid, contains multiple double bonds that can trap free radicals through resonance structures. Purified ethyl‐EPA reduces the incidence of cardiovascular events in high‐risk patients with elevated Lp(a) levels. Studies have shown that EPA inhibits the oxidation of all lipoprotein components and that EPA can significantly prevent the Lp(a)‐induced oxidative stress response of endothelial cells [98]. In addition to its antioxidant effects, EPA may modulate vascular inflammation by altering the profile of lipid mediators derived from polyunsaturated fatty acids [99]. Moreover, research has indicated that purified EPA can significantly and persistently enhance endothelium‐dependent relaxation in arterial rings by promoting endothelial NO production [100].

Proprotein convertase subtilisin/kexin type 9 (PCSK9) inhibitors, such as evolocumab and alirocumab, are well‐established lipid‐lowering agents that effectively reduce LDL and Lp(a) levels without raising safety concerns [101]. Lp(a) stimulation increased the protein and gene expression of PCSK9 by 20% and 44%, respectively, compared to the unstimulated control group [61]. The mechanism by which PCSK9 monoclonal antibodies reduce Lp(a) remains to be studied. Current research suggests that PCSK9 monoclonal antibodies lower circulating Lp(a) through mechanisms that may include enhanced receptor‐mediated clearance and, in some settings, reduced Lp(a) production [102]. The reduction of Lp(a) by PCSK9 inhibitors is usually limited and unstable, approximately 20% [103]. Studies have shown that PCSK9 inhibitors not only reduce blood lipid levels but also exert multiple effects. Zulkapli et al. demonstrated that both evolocumab and alirocumab reduced the protein expression of ICAM‐1 and E‐selectin, which are key adhesion molecules that mediate monocyte recruitment during early atherogenesis. Importantly, eNOS expression increased at high concentrations, particularly with evolocumab treatment. At lower doses, both inhibitors reduced the adhesion of monocytes to endothelial cells. The study concluded that PCSK9 inhibitors modulate endothelial cell activation, inflammation, and monocyte adhesion in Lp(a)‐induced HCAECs, and that the inhibitors play crucial roles in early atherosclerosis prevention [61].

Lipoprotein separation refers to the removal of lipoproteins containing apolipoprotein B, such as Lp(a). Lipoprotein separation has been shown to decrease the incidence of cardiovascular events in patients with high Lp(a) levels [104]. Compared with existing lipid‐lowering drugs, lipoprotein separation can significantly reduce Lp(a) levels to a greater extent. Research has indicated that Lp(a) levels decrease acutely 70%–80% following treatment, but the treatment effects could be reversed [105]. Recent research has shown that lipoprotein separation enhances endothelial function in patients with severe hypercholesterolaemia. A potential mechanism involves the enhancement of eNOS expression or activity in human endothelial cells, which increases NO production and improves arterial vasodilation. Conversely, lipoprotein separation leads to decreases in LOX‐1 and VCAM‐1 expression while enhancing vascular protection in human endothelial cells [106].

Although various treatment methods are currently available to reduce Lp(a) levels, improve endothelial cell function, and prevent atherosclerosis occurrence, many limitations still exist, and the mechanisms underlying these treatments remain unclear. Therefore, further research is required to elucidate them. (Table 1).

**TABLE 1:** Therapeutic strategies relevant to Lp(a) and endothelial function.

Therapy	Mechanism of action	Lp(a) reduction	Evidence on endothelial function	Clinical status	Major limitations/adverse effects
Oestrogen‐based therapy	Modulates hepatic apo(a) production and vascular oestrogen signalling	Approximately 20%	Preclinical evidence: attenuates Lp(a)‐induced endothelial adhesion‐molecule expression	Approved for selected menopausal indications; not indicated for Lp(a) lowering	Thromboembolic and hormone‐sensitive cancer risks; unsuitable solely for cardiovascular prevention
Niacin	Reduces hepatic apo(a) production	Approximately 20%–30%	Associations with improved endothelial function and reduced oxidative stress	Approved lipid‐modifying drug; not recommended specifically for Lp(a)	No incremental cardiovascular benefit with contemporary statin therapy; flushing, hepatotoxicity
EPA	Antioxidant and anti‐inflammatory effects	No consistent clinically meaningful reduction	Preclinical/vascular evidence of reduced lipoprotein oxidation and enhanced endothelial NO signalling	Approved for selected hypertriglyceridaemia/high‐risk populations; not an Lp(a)‐lowering therapy	Benefit cannot be attributed to Lp(a) lowering; bleeding and atrial fibrillation risks in susceptible patients
PCSK9 inhibitors (evolocumab, alirocumab)	Related to clearance and the production of Lp(a)	Approximately 20%	Preclinical evidence of reduced endothelial activation and monocyte adhesion in Lp(a)‐stimulated cells	Approved for LDL‐C lowering and ASCVD risk reduction	Modest and variable Lp(a) reduction; injection‐site reactions
Lipoprotein apheresis	Extracorporeal removal of apoB‐containing lipoproteins	Approximately 60%–80% acutely	Evidence of improved vasodilation and vasoprotective endothelial gene expression	Available for selected patients with severe, progressive disease	Invasive, costly, and transient in effect, requiring repeated procedures

## 6 Future Directions for Lp(a)–Endothelial Cell Research

Endothelial cells, monocytes, macrophages, and smooth muscle cells play essential roles in the progression of atherosclerosis. However, endothelial cells are the primary regulators, as they can induce a proinflammatory state, produce adhesion proteins, and secrete signalling molecules to facilitate cell interactions. The pathophysiology of endothelial cells has always been the focus of research on cardiovascular diseases. Mitigating endothelial dysfunction may be a viable approach for decreasing the risk of atherosclerosis and improving its prevention. Recent large‐scale genetic epidemiological studies have established a causal link between elevated Lp(a) levels and cardiovascular diseases, including coronary heart disease, aortic valve stenosis, and heart failure, increasing research interest in Lp(a). Furthermore, research has shown that Lp(a) promotes the development of ASCVDs and significantly affects the severity of coronary heart disease [107]. Lp(a) contributes to cardiovascular disease onset by disrupting endothelial homeostasis, which involves impairing the balance between endothelial damage and repair, promoting endothelial activation and dysfunction, and reducing the function of endothelial progenitor cells. Future research should elucidate the regulatory mechanisms of Lp(a) in cardiovascular disease and the role of endothelial cells to harness its therapeutic potential.

Large‐scale clinical trials should be conducted to determine whether Lp(a) can serve as a marker of endothelial dysfunction and a predictor of cardiovascular disease. However, according to the current clinical data, the median concentration of Lp(a) varies greatly. Beyond racial and ethnic population variations, standardising the current detection methods and establishing reference materials present significant challenges. To address the lack of standardisation, researchers have proposed converting Lp(a) measurements into molar concentrations [108]. After a consistent measurement standard is established, researchers can assess plasma Lp(a) levels in cardiovascular disease patients, along with the levels of endothelial cell activation markers and secreted inflammatory factors, for investigating their correlation. Endothelial biomarkers, including flow‐mediated dilation, NO‐dependent vasoreactivity, circulating adhesion molecules, endothelial microparticles, and vascular inflammatory imaging, may complement plasma Lp(a) measurements by reflecting biologically active vascular injury. Incorporating these endpoints into trials of Lp(a)‐targeted therapies may help determine whether profound Lp(a) reduction restores endothelial function and whether early vascular improvement predicts subsequent cardiovascular benefit. However, these biomarkers require prospective validation and should not currently replace established clinical risk assessment.

In addition, further exploration of how Lp(a) regulates changes in endothelial cell function is worthwhile. Currently, such research is extremely scarce. Although existing studies have shown that Lp(a) can regulate endothelial cell barrier function, inflammatory factor secretion, adhesion protein expression, eNOS dysfunction, oxidative stress, pyroptosis and other processes, the regulatory mechanisms, such as intracellular transcriptional regulation, molecular expression, and signal transduction, are still unclear. In vivo and in vitro experiments should be conducted to investigate the effects of Lp(a) on endothelial cells for identifying novel therapeutic targets, addressing endothelial cell dysfunction, and enhancing vascular lesion outcomes. This may be an effective strategy for reducing atherosclerosis development.

Lp(a) affects endothelial cell dysfunction and is involved in the process of atherosclerosis. Therefore, controlling Lp(a) levels from the source is also a treatment method for preventing cardiovascular disease. Genetic epidemiological studies have shown that lowering Lp(a) may have clinical benefits, but how to control it and the degree of reduction still need to be explored. At present, no drug therapy specifically targeting Lp(a) has been approved. Research has indicated that lifestyle modifications and widely used lipid‐lowering medications, such as statins and ezetimibe, do not effectively reduce Lp(a) levels. However, PCSK9 inhibitors can moderately decrease Lp(a) levels and delay the progression of coronary heart disease [109]. However, drug therapies, such as niacin, have unavoidable adverse reactions, and the use of special therapies, such as lipoprotein apheresis, is limited because of the validity period [110]. These represent the major challenges associated with the currently available therapies, but other treatment methods are still being explored. Clinical trials of nucleic acid therapies targeting the LPA gene, such as antisense oligonucleotides (ASO) and small interfering RNA (siRNA) drugs, are ongoing. However, extensive follow‐up and data analysis are necessary to confirm their clinical applicability [110]. Given the lack of treatment options, a better understanding of the origin and metabolism of Lp(a) should be gained, as well as of how it exerts its physiological and pathological functions. Such findings would aid in actively exploring new treatment methods and evaluating the safety, effectiveness, and sustained response to Lp(a)‐lowering therapies in clinical practice.

## 7 Conclusions

High Lp(a) levels and endothelial dysfunction significantly contribute to cardiovascular disease onset. In this review, we explored the generation and metabolism of Lp(a), as well as how Lp(a) can penetrate the endothelial barrier, induce oxidative stress, mediate a chronic proinflammatory state and trigger endothelial dysfunction (Figure 3). However, the molecular mechanisms involved remain to be explored. Additional in vitro and in vivo studies should be conducted given the lack of a safe and effective treatment for decreasing Lp(a) levels.

**FIGURE 3:**
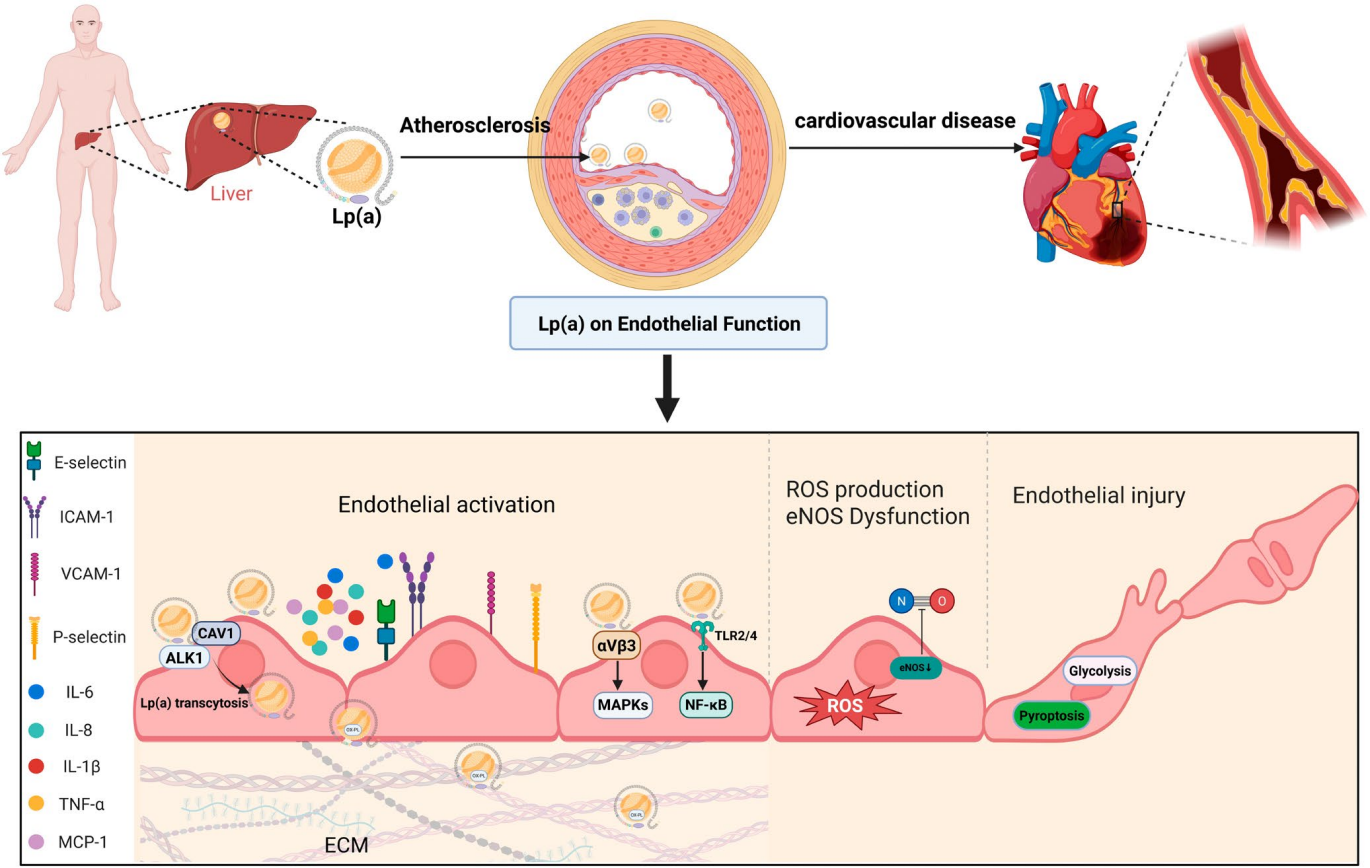
Lp(a) induces endothelial homeostatic imbalance: Lp(a) crosses the endothelial barrier, potentially through ALK1/CAV1‐associated transcytosis and accumulates in the subendothelial extracellular matrix. Lp(a) and oxidized Lp(a) promote endothelial activation through NF‐κB‐ and MAPK‐related signalling, thereby promoting an inflammatory milieu. This process stimulates the secretion of pro‐inflammatory cytokines such as IL‐1β, IL‐6, IL‐8, TNF‐α and MCP‐1. Furthermore, it enhances the expression of adhesion molecules, including ICAM‐1, E‐selectin, P‐selectin, and VCAM‐1, thereby facilitating intercellular signalling regulation. Concurrently, the production of ROS and impaired nitric oxide bioavailability disrupt vascular homeostasis. Additionally, Lp(a) contributes to endothelial damage through mechanisms involving pyroptosis and glycolysis.

## Author Contributions

**Xianghui Zhou:** writing – original draft, investigation. **Xiyuan Fang:** writing – review and editing. **Xin Zhou:** writing – review and editing. **Zhao Zhang:** investigation. **Yu Hu:** writing – review and editing. **Zhipeng Cheng:** writing – review and editing.

## Funding

This review was supported by the Program of National Key Research and Development Project of China (2023YFC2509500 to Zhipeng Cheng), the National Natural Science Foundation of China (No. 82500179 to Xianghui Zhou, No. 82470135 to Zhipeng Cheng, No. 82001026 for Xin Zhou), and Natural Science Foundation of Hubei Province (2026AFB279 to Xianghui Zhou).

## Conflicts of Interest

The authors declare no conflicts of interest.

## Data Availability

Data sharing not applicable to this article as no datasets were generated or analysed during the current study.
